# Application potential of *Klebsiella pneumoniae* in agriculture: biodegradation, plant growth promotion, and alleviation of biotic and abiotic stresses

**DOI:** 10.1515/biol-2025-1304

**Published:** 2026-04-20

**Authors:** Dan Liu, Baofeng Zhang, Min Liu, Xiaomei Song, Jingsheng Chen

**Affiliations:** Department of Landscape Engineering, Yangzhou Polytechnic Institute, Yangzhou 225127, China; Jiangsu Engineering Center for Modern Agricultural Machinery and Agronomy Technology, Yangzhou University, Yangzhou 225127, China; Department of Urban Design and Regeneration, Macau University of Science and Technology, Macau 999078, China; College of Horticulture and Landscape Architecture, Yangzhou University, Yangzhou 225009, China; College of Biology and Food Engineering, Chongqing Three Gorges University, Chongqing 404100, China

**Keywords:** *Klebsiella pneumoniae*, biodegradation, plant growth promoting, stress management

## Abstract

*Klebsiella pneumoniae*, a Gram-negative bacterium with a long history of research and diverse applications, has attracted increasing interest in the agricultural field in recent years. This review explores the ability of *K. pneumoniae* strains to degrade various pollutants, such as pesticides, veterinary drugs, biological toxins, exogenous contaminants, and agricultural wastes (e.g., herbicide, polycyclic aromatic hydrocarbons, dyes, cellulose, and lignin). In addition to its ability to promote plant growth via mineral solubilization, phytohormone production, and nitrogen fixation, *K. pneumoniae* helps plants mitigate biotic and abiotic stresses through the production of antagonistic substances and the induction of systemic resistance or tolerance. Given these multifunctional capabilities, its considerable promise for use in biofertilization, bioremediation, and biocontrol is increasingly recognized, and with further research, *K*. *pneumoniae* is expected to play a more prominent role in sustainable agricultural production.

## 1 Introduction

**Figure 1:**
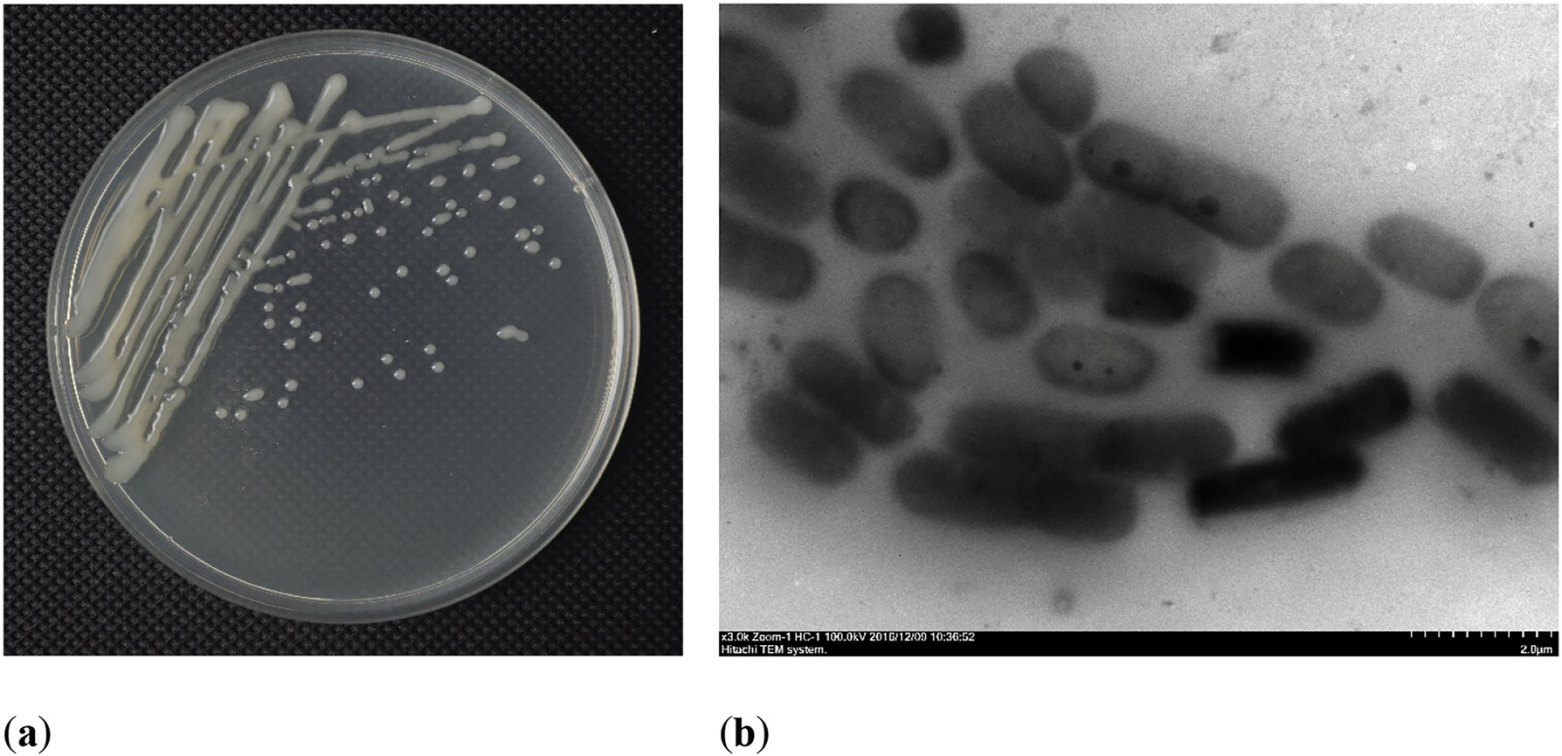
Morphological characteristics of *Klebsiella pneumoniae*. *K. pneumoniae* strain SnebYK was used in this figure. (a) Morphological characteristics of *K. pneumoniae* on NB plate; (b) *K. pneumoniae* was viewed under a transmission electron microscope. All images originated from the authors.

*Klebsiella pneumoniae* is a Gram-negative bacterium of the genus *Klebsiella* within the family Enterobacteriaceae. Cells measure approximately 0.3–1.5 μm in diameter by 0.6–6.0 μm in length, typically occurring as single cells, pairs, or short chains. It lacks flagella and spores but has a distinct capsule visible in direct smears and forms shiny, hemispherical colonies on nutrient-rich media (Figure 1). Its growth temperature ranges from 12 °C to 43 °C, with optimum growth observed at 37 °C [1], 2].

**Figure 2:**
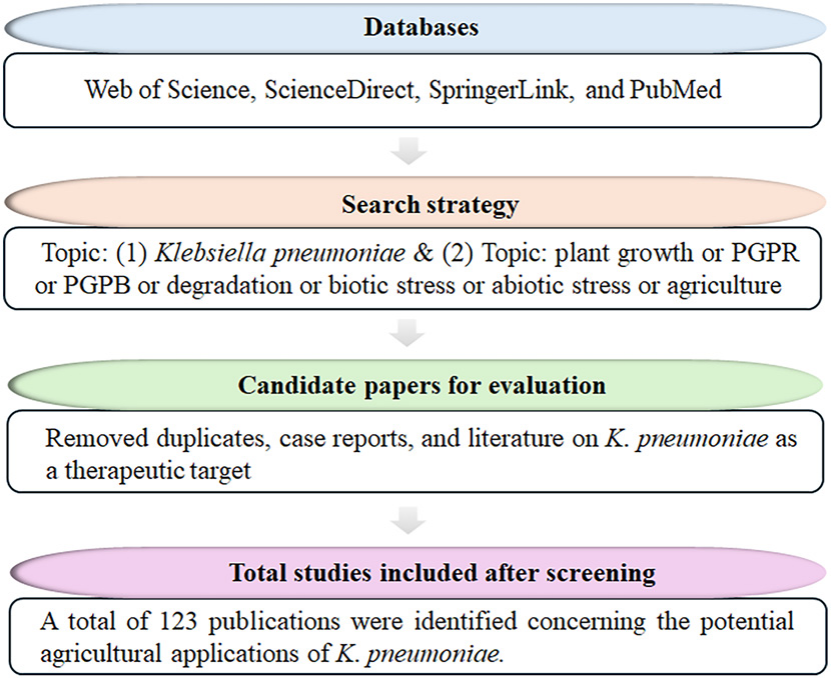
Schematic representation of the literature identification, screening, and inclusion process.

First reported by Friedlander in 1882, *K. pneumoniae* is known to cause pneumonia, meningitis, wound infections, and systemic sepsis and exhibits resistance to various antimicrobial agents [3]. In bioengineering, it is primarily employed for the production of 2,3-butanediol and 1,3-propylene glycol, as well as the synthesis of various other compounds, including ethanol, 3-hydroxypropionic acid, succinic acid, and lactic acid, offering significant industrial potential [4]. In contrast, the agricultural potential of *K*. *pneumoniae* remains underexplored. This review therefore aims to systematically investigate and delineate its prospective agricultural roles. To identify relevant publications, a broad literature search was conducted in the Web of Science, SpringerLink, PubMed, and ScienceDirect databases, targeting the period between 1971 and 2025. Key search terms incorporated “*K. pneumoniae*,” “plant growth,” “PGPR,” “PGPB,” “degradation,” as well as “biotic stress,” “abiotic stress,” and “agriculture.” The inclusion criteria specified English-language publications and one of the following types: original research articles, reviews, meta-analyses, or indexed book chapters. The exclusion criteria comprised duplicates, case reports, and any literature on *K*. *pneumoniae* as a therapeutic target. The screening process involved an initial review of titles, abstracts, and keywords, followed by a full-text assessment. This process culminated in the identification of 123 articles for final inclusion (Figure 2). To assess the agricultural application potential of *K*. *pneumoniae*, relevant data were compiled and analyzed, focusing on its roles in biodegradation, plant growth promotion, and the alleviation of both biotic and abiotic stresses.

## 2 Biodegradation activity

Although soil and water are crucial for agricultural production, they are being contaminated by various compounds due to industrial pollution and the excessive use of pesticides and veterinary drugs, degrading agricultural sustainability and posing risks to human health [5]. Moreover, the substantial accumulation of agricultural waste materials, including cellulose-based residues, constitutes a critically significant environmental challenge. The application of *K*. *pneumoniae* enables the degradation of various pollutants and agricultural waste, leading to a reduction in agricultural and environmental contamination and contributing to high-quality agricultural production.

### 2.1 Biodegradation of pesticides

Advancements in modern agriculture rely on the use of pesticides. However, residual pesticides pose a non-negligible pollution risk to the agricultural production. Utilizing microorganisms to degrade pesticides is regarded as an effective and environmentally friendly solution [6]. Among these, *K. pneumoniae* has emerged as a notable contributor to the degradation of various herbicides, pesticides, fungicides, and plant growth regulators (Table 1).

**Table 1:** *Klebsiella pneumoniae* strains used for pesticide degradation.

Target pesticide		Degradation strain	Original isolation source	Reference
Herbicide	Chlorinated *s*-triazines	A2	Wastewater plant sludge	[7]
	Atrazine	F-N1	River sediment	[8]
	Triazine-containing pollutants	*K. pneumoniae*	Polluted marine sediment	[9]
	S-metolachlor	GC s.B strain 1, GC s.B strain 2	Humus and soil	[10]
	Chlorimuron-ethyl	2N3	Sludge of industrial wastewater tank	[11]
Insecticide	Endosulfan	KE-1	Soil	[12]
	Endosulfan	*K. pneumoniae* JAS8 + fungal and bacterial strains	Endosulfan treated agricultural soils	[13]
	3-Phenoxybenzoic acid and pyrethroid	BPBA052	Soybean rhizosphere soil	[14]
	Deltamethrin	*K. pneumoniae* BPBA052 + *Acinetobacter junii* LH-1-1	Soybean rhizosphere soil	[15]
	Imidacloprid	BCH1	Agricultural soil contaminated with pesticide	[16]
	Cypermethrin	FCM82	Soil	[17]
	Chlorpyrifos	CP19	Municipal soil sediment	[18]
	Chlorantraniliprole	PPCO1	Farmgate fruits and vegetables	[19]
Fungicide	Tributyltin chloride	SD9	Surface water	[20]
Plant growth regulator	Paclobutrazol	M6 (MW228061)	Mango rhizosphere	[21]

In 1995, *K. pneumoniae* strain A2 was discovered to utilize 2-chloro-4,6-diamino-s-triazine, deethylsimazine, and deethylatrazine for nitrogen supply [7], while *K. pneumoniae* F-N1 was reported to degrade atrazine [8]. Photocatalytic treatments can degrade triazine-based contaminants such as Irgarol^®^ 1051 (cybutryne), C.I. Reactive Red 15, and simazine, whereas cyanuric acid, a byproduct of UV irradiation, cannot be fully mineralized. *K. pneumoniae* has been found to completely mineralize cyanuric acid within 24 h of incubation, facilitating more effective pollutant degradation [9]. Furthermore, the ability of *K*. *pneumoniae* strains to degrade Chlorimuron-Ethyl and S-Metolachlor has been confirmed [10], 11].

Beyond its established role in herbicide degradation, *K. pneumoniae* has shown exceptional performance in the degradation of insecticides. Endosulfan, a chlorinated cyclodiene insecticide commonly used on crops such as cotton, fruit trees, and tobacco, is highly toxic to aquatic organisms. Kwon et al. [12] revealed that endosulfan degradation by *K*. *pneumoniae* strain KE-1 occurs via a non-oxidative mechanism, thus, this process avoids the formation of toxic endosulfan sulfate. Additionally, *K. pneumoniae* strain JAS8 combined with other fungal and bacterial strains (*Halophilic bacterium* JAS4, *Enterobacter cloacae* JAS7, *Aspergillus tamarii* JAS9, *Lasiodiplodia* sp. JAS12, *Botryosphaeria laricina* JAS6 and *E. asburiae* JAS5) achieved efficient degradation of endosulfan at 1,000 mg/L in both aqueous media and soil [13], highlighting its potential for managing endosulfan contamination. Pyrethroid pesticides (PPs) are widely used to manage pest-related issues in both agricultural and public health. Although they are low in toxicity and highly effective, their acute toxicity to aquatic organisms and invertebrates remains a significant concern. Moreover, these residues can bioaccumulate in the food chain, ultimately threatening human health through dietary exposure. 3-Phenoxybenzoic acid (3-PBA) is commonly used as a biomarker to assess human exposure to pyrethroid insecticides. Tang et al. [14] isolated *K*. *pneumoniae* strain BPBA052 from soybean rhizosphere soil. This strain exhibited a 96.37 % degradation rate for 3-PBA (100 mg/L) over a 72-h incubation period. Strain BPBA052 also metabolized PPs and 3-PBA metabolites, and genes encoding degradative enzymes (*PROβ*, *LPH*, and *CatA*) were successfully cloned. Subsequent investigations revealed that a co-culture system containing *Acinetobacter junii* LH-1-1 and strain BPBA052 markedly improved the breakdown of both deltamethrin and 3-PBA [15]. Beyond the compounds previously mentioned, *K*. *pneumoniae* strains are also known to metabolize additional insecticides, including cypermethrin, imidacloprid, chlorpyrifos and chlorantraniliprole [16], [17], [18], [19] (Table 1).

Tributyltin chloride, commonly used as a fungicide, has harmful effects on the aquatic biota. *K. pneumoniae* strain SD9 isolated from surface water showed the capacity to transform tributyltin chloride into its less hazardous derivatives, namely dibutyltin dichloride and monobutyltin trichloride [20]. As a widely used plant growth regulator in agriculture and horticulture, paclobutrazol can damage soil microorganisms and plants owing to its prolonged presence in soil. *K. pneumoniae* strain M6 can degrade paclobutrazol, promote plant growth, and combat pathogenic fungi, making it a promising candidate for bioremediating paclobutrazol-contaminated soil [21].

### 2.2 Biodegradation of veterinary drugs

Pharmaceutical compounds such as ciprofloxacin, tetracycline, norfloxacin, ofloxacin, and diclofenac sodium are commonly used in animal and aquaculture health management but are not fully absorbed, leading to their release into soil and water and causing agricultural pollution [22], [23], [24]. Various *K. pneumoniae* strains and their microbial compositions have been shown to degrade these contaminants and reduce their toxicity (Table 2). Studies have explored possible degradation mechanisms by analyzing biodegradation metabolites. *K. pneumoniae* BSFLG-CIP1 may degrade ciprofloxacin through hydroxylation, piperazine ring substitution and cleavage, as well as breakdown of quinoline ring [23]. Similarly, *K. pneumoniae* WAH1 can promote the degradation of diclofenac sodium via hydroxylation, decarboxylation, and dechlorination [24]. The findings of Cheng et al. [25] demonstrate that *K. pneumoniae* even play a functional role in Teflaro (cephalosporin antibiotic) wastewater biodegradation via microbial fuel cell systems.

**Table 2:** *Klebsiella pneumoniae* strains used for the degradation of veterinary drugs and biological toxins.

Target pollutant		Degradation strain	Original isolation source	Reference
Veterinary drug	Ciprofloxacin	BSFLG-CIP1	Larval gut	[23]
	Diclofenac sodium	WAH1	Pharmaceutical sludge	[24]
	Tetracycline	TR5	Chicken manure mixture	[26]
	Norfloxacin, ofloxacin	*K. pneumoniae* (K2) + *Achromobacter* sp. (K3) + *Candida manassasensis* (K1) + *Trichosporon asahii* (K4)	Green compost	[27]
Biological toxin	Citrinin	NPUST-B11	Fruit farm soil free from pesticide contamination	[28]
	Zearalenone	GS7-1	Corn field	[29]
	Diaminopropionic acid	LPSR1	Rumen digesta	[30]
	Tannin	*K. pneumoniae*	Agricultural soil	[31]
	Tannic acid	*K. pneumoniae*	Goat feces	[32]
	Tannic acid	EO1	Lagoon water and mangrove soil samples	[33]
	Tannic acid	SEC-6	Silkworm excrement compost	[34]

### 2.3 Biodegradation of biological toxins

Certain highly potent biological toxins pose a contamination risk to a wide range of agricultural products, thus limiting their production. Zearalenone, which is commonly present in farm produce, food, and feed, is produced by *Fusarium* spp. and can cause acute and chronic poisoning in animals. As another important mycotoxin, citrinin frequently occurs during *Monascus* production and is highly toxic to microorganisms, humans, and animals. Under optimal conditions, *K. pneumoniae* strains GS7-1 and NPUST-B11 can completely degrade these toxins, with GS7-1 producing an enzyme involved in zearalenone degradation [28], 29]. Additionally, *K. pneumoniae* can degrade various toxic substances in plant-based feed. For instance, strain LPSR1 is capable of degrading diaminopropionic acid, a non-proteogenic amino acid that is toxic to ruminants and is found in the leguminous forage plant *Acacia angustissima* [30]. Species of the genus *Quercus* contain high levels of tannins, which reduce their suitability as animal feed by decreasing feed intake and nutrient digestibility and impairing rumen fermentation. *K. pneumoniae* has been shown to degrade tannins and phenolic compounds in two types of oak leaves, resulting in improved nutritional value in ruminant diets [31]. Multiple isolates of *K*. *pneumoniae* exhibit tannic acid-degrading activity [32], 33], and Shen et al. [34] discovered that *K. pneumoniae* strain SEC-6 could assist silkworms in breaking down tannic acid in mulberry leaves, improve feeding efficiency, and promote weight gain. These findings demonstrate that using *K. pneumoniae* is effective for the removal of various biological toxins (Table 2).

### 2.4 Biodegradation of exogenous pollutants

The intensification of industrial and human activities has released hazardous contaminants, including polycyclic aromatic hydrocarbons (PAHs), dyes, oil, and plastic waste, into ecosystems and agricultural systems, endangering both agricultural sustainability and public health. Several *K. pneumoniae* strains have been reported to contribute to degradation of these contaminants.

PAHs are stable organic pollutants widely distributed in terrestrial, atmospheric, and aquatic environments and are recognized for their toxic, mutagenic, and carcinogenic properties [35]. The United States Environmental Protection Agency (USEPA) has classified 16 PAHs as priority pollutants, seven of which are also included in China’s blacklist of priority pollutants [36], 37]. *K. pneumoniae* PL1 was found to degrade 63.4 % of pyrene and 55.8 % of benzo[*a*]-pyrene (BaP) within 10 d (20 mg L^−1^ pyrene and 10 mg L^−1^ BaP), indicating the improved degradation at pH 7 and in paddy soil [36]. Similarly, *K. pneumoniae* AWD5 degraded pyrene and promoted rice root growth in pyrene-contaminated soils [37]. *K. pneumoniae* produces extracellular polymeric substances (EPS) that facilitate PAH degradation. Premnath et al. [38] discovered that *K. pneumoniae* KY494861 produced maximum EPS growth and efficiently biodegraded PAHs, such as anthracene, acenaphthene, fluorene, and naphthalene, under optimized carbon/nitrogen ratios, temperature, and pH conditions.

Phenolic pollutants such as phenol, 2,4-dichlorophenol, bisphenol A, nitrophenol, and carbofuran phenol are commonly released into the environment through activities related to pesticides, pharmaceuticals, and the production of oil, coal, paper, and plastics [39], 40]. *K. pneumoniae* strains ZS01 [40], KZNSA [41], BYK-9 [42] and ATCC13883T [39] have been confirmed to biodegrade these phenolic contaminants (Table 3). Notably, polyurethane foam-immobilized *K. pneumoniae* ATCC13883T cells exhibited an enhanced degradation rate of carbofuran phenol [39]. Fang et al. [40] developed agar/carrageenan-Fe_3_O_4_-*K. pneumoniae* composite beads to further improve the phenol degradation rate. Microbial immobilization technology has enhanced the stability, longevity, and recyclability of *K. pneumoniae* to degrade phenolic pollutants, paving the way for future industrial applications.

**Table 3:** *Klebsiella pneumoniae* strains used for the degradation of exogenous contaminants.

Exogenous pollutant		Degradation strain	Original isolation source	Reference
Polycyclic aromatic hydrocarbon	Pyrene and benzo[*a*]-pyrene	PL1	Soil	[36]
	Pyrene	AWD5	Soil from industrial waste	[37]
	Naphthalene, anthracene, fluorene, and acenapthene	KY494861	Marine sources	[38]
	Pyrene	*K. pneumoniae*	Rhizospheric soil of *Scirpus triqueter*	[66]
Phenolic compound	Carbofuran phenol	ATCC13883T	Soil	[39]
	Phenol	ZS01 (CGMCC 16041)	Laboratory	[40]
	2,4-Dichlorophenol	KZNSA (*Kp*KZNSA)	Sludge from wastewater treatment plant	[41]
	Bisphenol A	BYK-9	Petrochemical wastewater	[42]
	Nitrophenol	*K*. *pneumoniae*	Wetland water	[67]
Dye	Methylene blue	UMTFA1 (EK)	Contaminated soil	[44]
	Congo red, malachite green	K2	Seawater	[47]
	Disperse blue-284	GM-04	Textile industrial effluent	[49]
	Methyl red	RS-13	Dye-contaminated sludge	[68]
	Malachite green	WA-1	Contaminated sediments	[69]
	Reactive blue 214, reactive yellow 145, and reactive red 195	MW815592	Textile waste	[70]
	Crystal violet	ED2	Textile industrial effluent sediment samples	[71]
Oil	Diesel	Kp (AR0139)	Activated sludge of petroleum refinery	[53]
	Crude oil	ATCC13883	Drilling fluid	[51]
	Crude oil	K05	Contaminated water	[54]
	Crude oil	SKBA6	Oil refinery plant (contaminated soil, sludge deposition, wastewater)	[55]
	Petrol	YSA-9	Soil contaminated with petroleum	[72]
	Kerosene	*K*. *pneumoniae* sp. *pneumoniae*	Roots of barley	[73]
Explosive	2,4,6-Trinitrotoluene (TNT)	SC1 K1	Soil containing TNT residues	[56]
	TNT	SU K3	TNT-polluted waste pink water	[57]
	Hexahydro-1,3,5-trinitro-1,3,5-triazine	SCZ-1	Anaerobic sludge	[58]
Plastic	Polyethylene	PE-S2-12bb (PS)	Soil from municipal waste landfill	[59]
	High-density polyethylene	CH001 (MF399051)	Plastic waste dumpsite	[60]
Other pollutant	Cyanides	Kp2	-	[61]
	Benzonitrile and Butyronitrile	NCTR1	Wastewater facility	[62]
	Thiocyanate	*K*. *pneumoniae* (KMK-L) + *Ralstonia* sp. (KMK-S)	Soil from the effluent disposal site of zinc cyanide electroplating industry	[63]
	Acrylonitrile	No. 1 (GU903318)	Domestic wastewater	[64]
	Acrylonitrile	*K*. *pneumoniae*	Industrial sewage	[65]

Every year, a significant volume of high-color wastewater is discharged into the environment by factories, primarily from industries such as textiles, printing, and food processing [43], [44], [45]. These dye pollutants alter the pH of water, diminish oxygen solubility, and impair light utilization capacity of aquatic plants [46], 47]. In addition, they can decrease soil fertility, hinder seed germination and growth, and ultimately reduce plant productivity [48], 49]. Several *K. pneumoniae* strains are capable of decolorizing and breaking down diverse synthetic dyes such as azo and triphenylmethane dyes (Table 3). Notably, the *K. pneumoniae* strain K2 decolorizes Direct Red 28 and Basic Green 4. Furthermore, when the degraded solution was used to treat wheat and sorghum, neither seed germination nor seedling development (shoot and root elongation) was affected, suggesting the detoxifying capability of strain K2 [47].

Approximately 600,000 tons of pure oil is estimated to leak into the environment annually [50], 51], making petroleum-derived hydrocarbons among the most widespread chemical contaminants in both soil and water systems [52], 53]. *K. pneumoniae* strains, such as ATCC13883, K05, and SKBA6, have been confirmed to use crude oil as their only carbon substrate, achieving effective biodegradation [51], 54], 55]. The K05 strain, in particular, was identified to possess *alkB1* and *nahAc* genes, enabling it to degrade both aliphatic and aromatic hydrocarbons.

Certain *K. pneumoniae* strains demonstrate notable efficacy in the bioremediation of diverse contaminants, including explosives such as 2,4,6-trinitrotoluene and hexahydro-1,3,5-trinitro-1,3,5-triazine [56], [57], [58], plastics such as polyethylene and high-density polyethylene [59], 60], and cyanides such as potassium cyanide, potassium hexacyanoferrate (II) trihydrate, and sodium ferrocyanide decahydrate [61]. Furthermore, they possess the capability to metabolize compounds (e.g., butyronitrile, thiocyanate, benzonitrile, and acrylonitrile) [62], [63], [64], [65], further underscoring their potential utility in bioremediation strategies for diverse contaminant types.

### 2.5 Biodegradation of cellulose and lignin

Cellulose, a linear homopolysaccharide of β-glucose, and lignin, composed of p‐hydroxyphenyl, guaiacyl, and syringyl monomers, are the first and second most abundant biopolymers in nature, respectively, primarily identified in plants. The effective utilization of important agricultural resources has garnered significant attention [74], 75]. However, in most agricultural production systems, cellulosic residues are typically disposed of through biomass combustion, which not only wastes resources but also causes environmental pollution [74], 76]. The biodegradation of cellulose and lignin by bacteria offers an energy-saving and environmentally friendly alternative [75]. *K*. *pneumoniae* has been repeatedly identified as capable of degrading cellulose and lignin [74], contributing to more efficient agricultural waste utilization and treatment of wastewater from paper mills.

*K*. *pneumoniae* Y7-3, isolated from ovine rumen microbiota, is a cellulose-degrading bacterium capable of producing high-yield hydrogen through anaerobic fermentation using corn straw as a carbon source [77]. *K*. *pneumoniae* B-11, which can be extracted from buffalo rumen fluid, has lignin-degrading abilities and provides insights into buffalo tolerance to roughage [75]. In paper mill wastewater containing residual lignin and other persistent organic pollutants, bacterial consortia, including lignin-degrading *K*. *pneumoniae* strains (GU193981 and GU193983), can effectively degrade and decolorize lignin, reducing pollution parameters such as chemical and biological oxygen demand. The detoxifying effect of these strains was confirmed by seed germination tests on *Phaseolus aureus*, indicating the safety of the treated wastewater [78], 79]. Using a metagenomic sequence-guided strategy, Tao et al. [80] identified *K*. *pneumoniae* ATCC 35657 and associated enzymes responsible for lignin degradation in fermented tobacco leaves. Specifically, their work revealed 51 genes encoding laccases and related multicopper oxidases.

The broad biodegradation profile of *K*. *pneumoniae* targets key recalcitrant structural motifs rather than specific pollutant classes. It demonstrates high efficacy in cleaving aromatic and polycyclic aromatic backbones, which constitute the core of many persistent contaminants, alongside robust activity against halogenated organics and various heterocyclic and nitrogenous compounds. This versatility correlates with a conserved set of enzymatic strategies, as illustrated in Table 4. Oxidative cleavage, facilitated by diverse oxygenases (as evidenced by the degradation of lignin, pharmaceuticals, and PAHs), and hydrolysis, mediated by specific esterases, amidases, and other hydrolases (as shown for pesticides and nitriles), emerge as the dominant and complementary pathways. These core mechanisms, supported by genomic evidence for extensive oxidative potential in strains like Kp342, KpC4, and augmented by accessory processes like dehalogenation and extracellular enzymatic action, underpin the bacterium’s adaptable and multi-functional degradation capacity across diverse environmental matrices.

**Table 4:** Key degradation mechanisms and determinants reported for *Klebsiella pneumoniae*.

Target pollutant	Degradation strain	Proposed mechanism or key determinant	Reference
Chlorimuron-ethyl	2N3	Hydrolases expressed by open reading frames (ORFs) 0934 and 0492	[11]
Endosulfan	KE-1	Non-oxidative pathway	[12]
3-Phenoxybenzoic	BPBA052	*LPH* (encoding phenol hydroxylase), *CatA* (encoding catechol 1,2-dioxygenase), and *PROβ* (encoding protocatechuate 3,4-dioxygenase)	[14]
Ciprofloxacin	BSFLG-CIP1	Hydroxylation, piperazine ring substitution and cleavage, and quinoline ring cleavage	[23]
Diclofenac sodium	WAH1	Hydroxylation, decarboxylation, and dechlorination reactions	[24]
Zearalenone	GS7-1	Extracellular enzymatic degradation (mediated by enzymes in the cell-free supernatant)	[29]
Crude oil	ATCC13883	Multi-enzymatic degradation mediated by both chromosomal/plasmid-encoded systems and secreted extracellular enzymes	[51]
Crude oil	K05	Gene-specific enzymatic degradation (via *alkB1* and *nahAc*)	[54]
Benzonitrile and Butyronitrile	NCTR1	Thermostable amidase	[62]
Lignin	ATCC 35657	Multicopper oxidase-mediated oxidative degradation (genomic evidence: 51 laccase/MCO genes)	[80]
Methyl orange	*K*. *pneumoniae*	Purified azoreductase enzyme	[81]

## 3 Plant growth promotion attributes

Nitrogen is a key nutrient that can limit plant growth in agriculture [82], and the major goal of nitrogen fixation research is to enable non-leguminous plants to fix nitrogen [83]. Nitrogen-fixing microorganisms such as *K*. *pneumoniae* can supply this nutrient to various non-legume plants [84]. *K*. *pneumoniae* 342 (Kp342) was demonstrated to alleviate nitrogen (N) deficiency symptoms in wheat. While establishing comparable colonization patterns and population sizes, Kp342 inoculation yielded significantly higher total nitrogen and nitrogen concentration in wheat relative to its nitrogen-fixation-deficient mutant or uninoculated controls. Despite conclusive evidence from ^15^N isotope dilution and nitrogenase reductase production, the nitrogen-fixation trait of Kp342 is cultivar-specific (cv. Trenton) [83]. Consequently, the total nitrogen increase in wild-type inoculations is likely the result of a combination of mechanisms, including but not limited to biological nitrogen fixation. Other mechanisms such as enhanced nutrient uptake efficiency, modification of root architecture, or broader microbiome-mediated effects likely contribute synergistically. Furthermore, the expression of related nitrogen uptake and assimilation genes in wheat merits thorough examination.

*K. pneumoniae* possesses nitrogen fixation genes organized into a cluster of 17 consecutive genes, classified into seven or eight operons that encode nitrogenase. Owing to its similarity to *Escherichia coli*, *K. pneumoniae* is the most extensively studied nitrogen-fixing species [85], and its nitrogenase has been successfully transferred into engineered strains [82]. The presence of the *nif* gene cluster indicates the genetic potential for nitrogen fixation, while functional activity requires confirmation through measures of gene expression, nitrogenase activity, or *in planta* assays. Rueda-Puente et al. [86] isolated a nitrogen-fixing strain of *K. pneumoniae* from the rhizosphere of the halophyte *Salicornia bigelovii*. This bacterium significantly promoted the growth of wild-type *S. bigelovii*, as evidenced by biomass data collected during germination and early seedling development. However, the researchers did not evaluate the efficacy of this strain when applied to non-halophytic plants.

As a nitrogen-fixing bacterium, *K. pneumoniae* can effectively colonize various plants. The *K. pneumoniae* NG14 strain, which harbors the *nifH* gene, was isolated from rice roots and demonstrated efficient colonization of both the root surface and the vascular tissues [87]. Dong et al. [84] further demonstrated that Kp342 colonized the roots and rhizospheres of five hosts (*Medicago sativa*, *M*. *truncatula*, *Arabidopsis thaliana*, *Oryza sativa*, and *Triticum aestivum*), with endophytic colonization in monocotyledonous plants being approximately 100-fold higher than in dicotyledonous plants, as quantified by colony-forming units (CFU) per gram of root tissue. The underlying mechanisms for this disparity remain speculative. Fluorescence-based imaging showed that Kp342 cells preferentially accumulate at the junction sites of lateral roots. The authors postulate that this may be attributed to either the larger apoplastic volume in monocots providing a more extensive habitat for endophytes, or to the production of specific exudates by these plants. In summary, bidirectional genetic compatibility functions as a central mechanism driving the active establishment of endophytic colonization.

**Figure 3:**
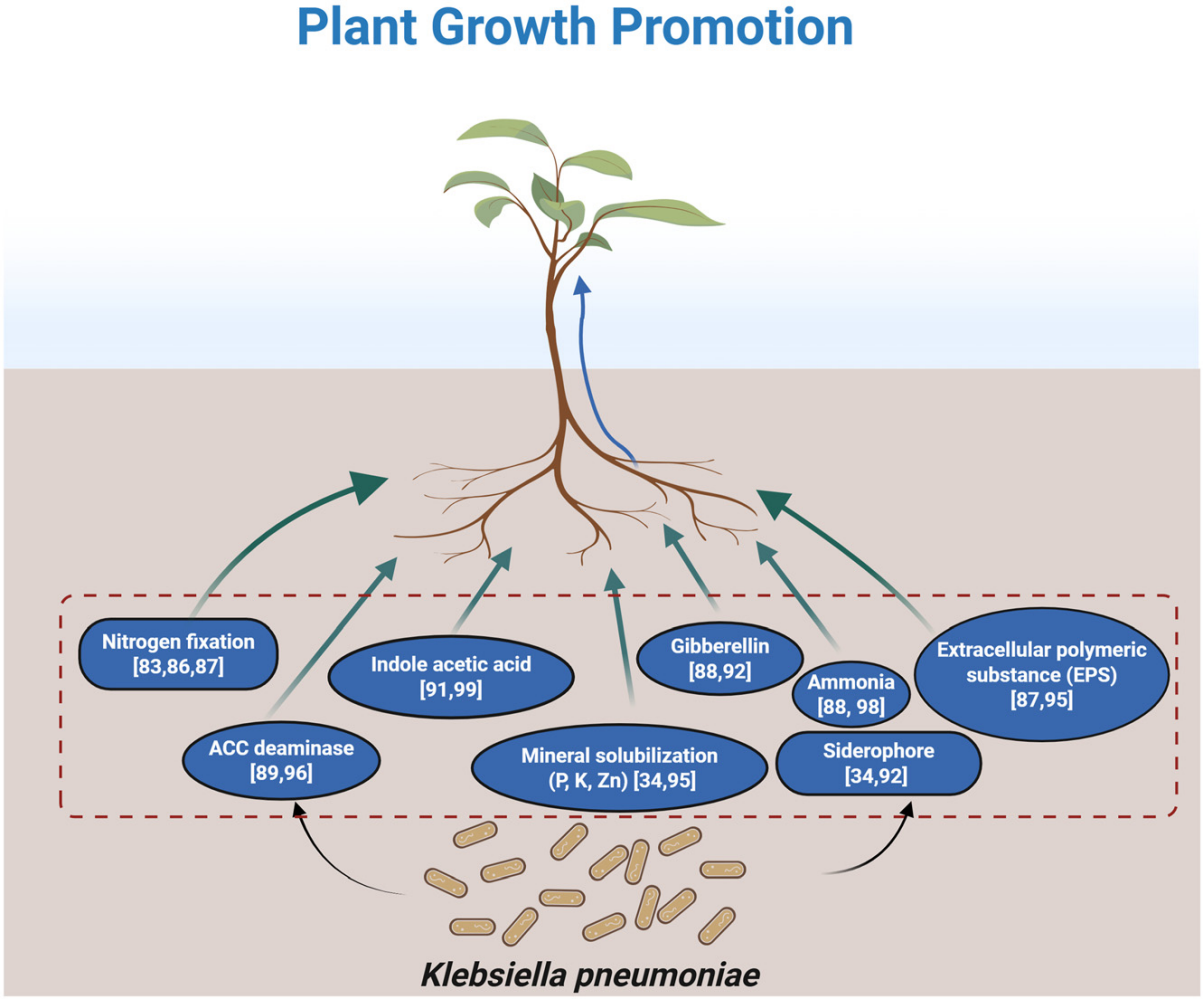
Schematic representation of the plant growth-promoting properties of *Klebsiella pneumoniae* (figure was created using BioRender.com). References supporting the illustrated mechanisms are provided directly in the figure.

In addition to its nitrogen-fixing capability, *K. pneumoniae* exhibits various plant growth-promoting properties, including the production of ammonia, siderophores, EPS, phytohormones [88], 1-amino cyclopropane carboxylate (ACC) deaminase [89], and mineral solubilization [90], making it a potential plant growth-promoting bacterium (PGPB) (Figure 3). From the wheat rhizosphere, Sachdev et al. [91] obtained nine strains of *K. pneumoniae*. Among these, six were found to secrete indole acetic acid (IAA), and strain K8 showed the highest production level at 27.5 mg/L. Germination tests indicated that strains K11 and K42 increased the root length in moth beans, whereas pot experiments demonstrated that all six IAA-producing strains significantly improved the root length and shoot height in wheat seedlings. In addition, the strain *Klebsiella* sp. SBP-8 was originally isolated from the rhizosphere of *Sorghum bicolor*. It had a 16S rRNA gene with 96 % similarity to *K. pneumoniae* HKG219, exhibited phosphorus-solubilizing activity, and produced phytohormones such as IAA and gibberellin, as well as siderophores and ammonia. SBP-8 also maintained high ACC deaminase activity at 6 % NaCl, with the functional gene *AcdS* encoding ACC deaminase identified in its genomic DNA [92]. Four *K. pneumoniae* strains (KW7-S06, KW7-S22, KW7-S27, and KW7-S33) were isolated from rice as endophytic diazotrophic bacteria and PGPB, all of which contain the *nifH* gene and exhibit plant growth-promoting traits that could enhance rice growth. Plasma technology enhances the proliferation and vitality of KW7-S06, enabling it to adhere more effectively to rice seed surfaces, resulting in improved germination rates for both rice and barley, as well as promoting rice growth [93], 94]. Additionally, a positive effect of the *K. pneumoniae* PRB-8 strain was observed, manifested as increased germination efficiency and an elevated vigor index in rice seedlings. Seed priming with *K. pneumoniae* PRB-8 led to a significant enhancement in the growth of rice, as evidenced by increased root length and fresh biomass. This growth-promoting effect is attributable to the strain’s multiple beneficial traits, including the solubilization of phosphate, potassium, and zinc, along with robust biofilm formation [95]. A proteomics study by Liu et al. [87] on *K. pneumoniae* NG14 before and after biofilm formation identified 28 significantly altered proteins, including the upregulation of the membrane pore protein OmpC, which is associated with osmotic stress resistance. *K. pneumoniae* P7, characterized by high ACC deaminase activity, was shown to promote cluster root formation in white lupin under low-phosphorus conditions through ethylene-mediated signaling [96]. Furthermore, *K. pneumoniae* I109 demonstrated remarkable efficacy in enhancing seagrass (*Zostera marina*) growth by restructuring rhizosphere bacterial communities, leading to a significant enrichment of functional groups involved in nitrogen fixation and aromatic compound degradation [97]. Collectively, these studies provide valuable insights into the plant growth-promoting effects and mechanisms of *K. pneumoniae* as PGPB.

Most strains of *K. pneumoniae* have been applied to monocotyledonous plants such as rice, wheat, and barley. However, the PGPB strain *K. pneumoniae* SnebYK also demonstrated plant growth-promoting effects on dicotyledonous plants, particularly soybeans, by significantly increasing the fresh weight, total root length, surface area, and root volume of soybean seedlings [98]. Similarly, inoculating cowpeas with the endophytic bacterium *K*. *pneumoniae* MEBAphS1 enhanced both above- and below-ground growth parameters, alongside a significant increase in chlorophyll B content [99]. Kumar et al. [100] developed an environmentally friendly, degradable surfactant using *K*. *pneumoniae* ssp. *ozaenae* BK34, which produced a biosurfactant, BS34, from butter waste. Identified as a surfactin-like glycolipopeptide, BS34 is non-toxic to plants and has been shown to promote seed germination, growth, and yield attributes in chickpea crops.

## 4 Abiotic and biotic stress management

**Figure 4:**
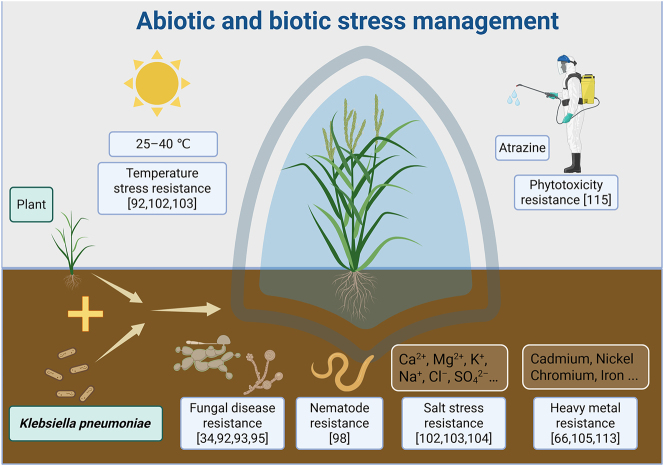
Role of *Klebsiella pneumoniae* in managing biotic and abiotic stresses (figure was created using BioRender.com). References supporting the illustrated mechanisms are provided directly in the figure.

*In vitro* experiments have demonstrated that *K. pneumoniae* strains exhibit broad-spectrum antifungal activity against plant pathogens (Figure 4), including *Rhizoctonia solani*, *Alternaria alternata*, *Aspergillus flavus*, *Sclerotium rolfsii*, *Fusarium oxysporum*, *Macrophomina phaselina*, *Botrytis cinerea*, *Botryosphaeria dothidea*, *Fusarium graminearum*, *Pestalotiopsis chamaeropis, Didymella bellidis*, and *Ralstonia solanacearum* [34], 92], 93], 95]. This antifungal activity may be related to the production of chitinase, hydrogen cyanide (HCN), and other inhibitory substances by *K. pneumoniae* [92], 95], as well as the presence of antibiotic biosynthesis-related genes, such as *PKS I* (polyketide synthase), *SrfC* (surfactin synthase) and *NRPS* (nonribosomal peptide synthetase) in *K. pneumoniae* [34]. In pot experiments, the disease index was significantly reduced in rice plants challenged with *R. solani* and *F. oxysporum* following treatment with *K. pneumoniae* strain PRB-8. This effect is likely attributable to an enhanced defense response, characterized by elevated activities of key enzymes including phenylalanine ammonia-lyase, β-1,3-glucanase, polyphenol oxidase, superoxide dismutase, catalase, and peroxidase, all of which are crucial for plant protection against pathogens [95].

Additionally, as a PGPB strain, *K. pneumoniae* can induce systemic resistance (ISR) against biotic stressors such as fungi and nematodes (Figure 4). Ji et al. [93] isolated the *K. pneumoniae* strain KW7-S06, which not only promoted the rice growth but also induced the resistance to *R. solani* and *F. oxysporum* following seed treatment. Biochemical assays confirmed acetoin synthesis by strain KW7-S06, with this volatile compound serving as a known ISR elicitor that reduced disease symptoms in *Arabidopsis* [101]. Split-root experiments confirmed that *K. pneumoniae* SnebYK controls soybean cyst nematodes (SCN) by inducing systemic resistance, inhibiting the invasion of juveniles, and suppressing nematode development in the roots. The SnebYK-mediated resistance was associated with increased expression of defense genes *PR1*, *PR2*, *PR5*, and *PDF1.2*, indicating activation of the salicylic acid and jasmonic acid/ethylene signaling pathways. Two years of field experiments have demonstrated the effectiveness of SnebYK in controlling SCN, highlighting the potential of *K*. *pneumoniae* as a biocontrol agent [98].

Moreover, *K. pneumoniae* is highly effective at mitigating various abiotic stresses in plants (Figure 4). According to studies by Rueda-Puente et al. [102], 103], inoculation with *K*. *pneumoniae* significantly enhanced key growth parameters, including germination rate, plant height, root elongation, and both dry and fresh biomass, in *S. bigelovii* and *Capsicum annuum* var. *aviculare* under saline conditions compared to non-inoculated controls. Noman et al. [104] used *K*. *pneumoniae* strain NST2 to synthesize copper nanoparticles (CuNPs). Application of these CuNPs to saline soil at a rate of 100 mg kg^−1^ not only increased maize biomass but also reduced lipid peroxidation and oxidative damage. This was achieved through promotion of antioxidant activity, decrease in cellular reactive oxygen species levels, and reduction in the accumulation of sodium ions (Na^+^) and chloride ions (Cl^−^).

*K*. *pneumoniae* performs well in alleviating heavy metal stress [105], 106]. Chandwani et al. [107] isolated 250 iron-tolerant PGPB, among which *K*. *pneumoniae* IMN17, upon inoculation into rice, increased plant biomass and alleviated iron stress by facilitating iron sequestration from the soil. The rhizobacterium *K. pneumoniae*, isolated from *Scirpus triqueter*, was found to enhance plant resistance in soils co-polluted with pyrene and nickel, and increase fluorescein diacetate activity [66]. Additionally, biogenic copper nanoparticles produced by *K*. *pneumoniae* SN35 can improve wheat growth, biomass, and cellular antioxidant content while immobilizing chromium (Cr) in the soil, resulting in the inhibition of its translocation to aerial plant components and alleviating oxidative stress [108]. Similar mechanisms were observed in *Brassica juncea* under a combined treatment with zinc oxide nanoparticles and *Klebsiella* sp. SBP-8 that alleviated Cr toxicity [109]. *K*. *pneumoniae* displays remarkable arsenic tolerance, with strains RnASA11 and ASBT-KP1 identified as arsenic-resistant isolates. *K*. *pneumoniae* RnASA11 contains genes related to resistance to heavy metals and oxidative stress, antimicrobial compound production, and plant growth promotion. It demonstrates efficient arsenic assimilation capabilities and tolerance to both arsenate and arsenite. Rhizosphere inoculation with RnASA11 significantly reduced arsenic uptake in *Vigna radiata* and *O. sativa*, establishing a promising bioremediation strategy for arsenic-contaminated soils [110]. *K*. *pneumoniae* also exhibits high tolerance for cadmium (Cd) [2], 111]. Inoculation of *Pennisetum giganteum* with a microbial consortium consisting of *K. pneumoniae* kpa (A4), *E. cloacae* RCB980 (A3), and *Klebsiella* sp. XT-2 (A7) enhanced plant growth and facilitated the phytoremediation of Cd-contaminated soil [112]. *K. pneumoniae* MCC3091 showed similar Cd toxicity mitigation effects in rice [113]. Furthermore, the lead (Pb)-resistant strain *K*. *pneumoniae* PbS3A2 significantly enhanced fish survival and growth parameters in *Labeo rohita* while mitigating the adverse effects of Pb toxicity in aquaculture systems, suggesting broad applicability of this species for heavy metal remediation in various environments [114].

*K. pneumoniae* also protects the plants from abiotic stress through induced systemic tolerance. The PGPB strain *Klebsiella* sp. SBP-8 alleviates growth inhibition in wheat under salt and temperature stress by modulating the K^+^/Na^+^ ratio, thereby improving salt tolerance [92]. Chen et al. [115] reported that soybean seeds coated with the *K*. *pneumoniae* strain SnebYK developed resistance to atrazine herbicide damage, with the fermentation liquid retaining its inducing activity even after sterilization. Research into the induction mechanism of SnebYK has revealed that it can upregulate the photosynthesis-related proteins such as ribulose-1,5-bisphosphate carboxylase/oxygenase (RubisCO) large subunit, antioxidant enzymes such as 2-Cys-peroxiredoxin-BAS1-like protein, lycopene e-cyclase, and detoxification enzymes like glutathione S-transferase F9. These mechanisms help mitigate atrazine phytotoxicity in soybeans and ensure normal plant growth.

## 5 Biosafety concerns, risk assessment, and regulatory challenges

Despite the considerable agricultural potential demonstrated by *K*. *pneumoniae*, its role as a human pathogen cannot be overlooked, particularly given its intrinsic virulence and significant antibiotic resistance. Direct infection risks aside, *K*. *pneumoniae* is a recognized reservoir for resistance plasmids (e.g., KPC, NDM-1) [1]. This characteristic raises serious biosecurity concerns regarding the use of bioinoculants, as these resistance determinants can propagate within soil microbial communities. Through horizontal gene transfer, such genes may disseminate to indigenous soil bacteria, potentially fostering the emergence of multi-resistant environmental “superbugs” [116].

Pathogenicity evaluations and acute toxicity tests demonstrated that *K*. *pneumoniae* strains PRB-8 [95], PbS3A2 [110] and kpa (A4) [117] were safe and nonpathogenic, which implies that pathogenicity may vary between strains. Genomic comparisons reveal distinct evolutionary lineages between agricultural and clinical strains of *K*. *pneumoniae*. A key differentiating feature is the functional nitrogen fixation gene cluster present in plant-beneficial isolates like strain 342, which is notably absent in the clinical reference strain MGH78578. Further genomic divergence is observed in genes mediating host interaction, such as those involved in adhesion, secretion, transport, and regulatory signaling, reflecting niche adaptation. Importantly, although strain 342 carries predicted virulence and antibiotic resistance determinants, *in vivo* pathogenicity assessments, such as murine model studies, demonstrate significantly attenuated disease potential compared to clinical isolates [118].

Building upon the genomic distinctions and attenuated virulence observed in specialized plant-associated strains, it is critical to acknowledge that virulence potential in *K*. *pneumoniae* is not universally linked to isolation source. Empirical evidence supports this, as comparative studies utilizing animal models (e.g., for urinary tract infection and intestinal colonization) and epithelial cell adhesion-invasion assays have shown that environmental isolates (such as those from surface water) can possess virulence comparable to clinical strains. While considerable strain-to-strain variation exists, these findings underscore that environmental origin alone does not guarantee avirulence, and risk assessment must account for this potential [119].

*K*. *pneumoniae* can naturally occur in the food supply. A relevant genomic surveillance study characterized strains isolated from ready-to-eat vegetables [120]. Analysis revealed that while *K*. *pneumoniae* was present, most isolates harbored few or no detectable antimicrobial resistance genes against critical drug classes. This indicates that the baseline antimicrobial resistance (AMR) risk from naturally occurring strains in such foods may currently be low. However, it is critical to note that this assessment does not reflect the scenario following the intentional application of *K*. *pneumoniae* as a bioinoculant. To date, no dedicated studies have evaluated the AMR profile or the potential for resistance gene enrichment in crops, vegetables, or fruits harvested from systems where *K*. *pneumoniae* has been actively introduced as a bioinoculant or agricultural amendment. This constitutes a significant knowledge gap. Therefore, any future deployment must be preceded by rigorous, targeted risk assessments that specifically address this concern, alongside the continued emphasis on stringent hygiene practices across the food production continuum.

The ‘One Health’ concept underscores the interconnectedness of human, plant, and environmental health through shared microbiomes [1]. Within this framework, the potential use of *K*. *pneumoniae*, a known vector for AMR genes, in agriculture necessitates a rigorous, integrated surveillance and management strategy to align with global AMR mitigation efforts. This strategy should include: (i) pre-application genomic screening of candidate strains to exclude those harboring mobile resistance elements or critical virulence factors; (ii) field-scale monitoring to track inoculant persistence, colonization, and potential gene transfer; and (iii) the development of coordinated, cross-sectoral (agricultural, environmental, health) policies that incorporate AMR risk assessment for agricultural bioinoculants into national and international regulatory frameworks [116].

## 6 Prospects and future directions

Recently, research on *K. pneumoniae* in agriculture has gained increasing attention. While numerous bacterial genera, such as *Bacillus*, *Pseudomonas*, and *Azospirillum* [121], 122], are well-established and safer alternatives for agricultural biostimulation and bioremediation, *K*. *pneumoniae* presents a compelling, albeit more complex, subject for scientific inquiry and potential application. It is important to recognize that certain environmental and plant-associated strains of *K*. *pneumoniae* demonstrate a combination of metabolic versatility, stress tolerance, and endophytic competence that is not universally paralleled. Equally important is the organism’s role in fundamental science, as *K*. *pneumoniae* critically helps bridge our understanding of the evolutionary continuum from mutualism to pathogenicity. This has led to increased research attention focused on exploring its agricultural potential. Initially, since the 18th century, the focus has been primarily on its nitrogen-fixing capabilities [123], whereas in the 21st century, greater emphasis has been placed on its biodegradation, plant growth-promoting, and biocontrol effects [124]. Research is steadily advancing in both breadth and depth. However, several key issues remain to be resolved.

The mechanisms underlying its degradation capacity, plant growth promotion, and biocontrol activity remain insufficiently explored. Most studies have focused on observed phenomena, leaving crucial mechanisms such as degradation pathways, induction pathways, and types of elicitors unclear. With the development of whole-genome sequencing technology, genes related to iron uptake, HCN biosynthesis, glycosylase activity, PAH utilization and heavy metal resistance, such as 342, SSN1, AWD5 and kpa (A4), have been identified in PGPB *K. pneumoniae* strains, providing significant insights for future studies on these mechanisms [117], 118], 125], 126].

While the compiled laboratory data conclusively establish the intrinsic metabolic and plant-growth-promoting potential of *K. pneumoniae*, a significant translational gap impedes its effective deployment in agricultural settings. The foremost challenge to scalability lies in the predominant reliance on evidence from controlled *in vitro* and greenhouse studies, which inherently simplify the multifaceted biotic and abiotic dynamics of field soil ecosystems. Critically, empirical data from field trials involving the deliberate application of *K. pneumoniae* as a bioinoculant remain exceptionally limited [127], which constitutes a major knowledge gap. This is possibly due to concerns over its potential pathogenicity to humans, as well as the pathogenicity of certain strains to crops such as maize [128], 129]. This scarcity of *in situ* validation hinders the prediction of essential performance criteria, including the ecological competitiveness of introduced strains against indigenous microbial communities for resources and niches, the functional persistence of their pollutant degradation or plant-beneficial activities under variable environmental conditions, and the context-dependent efficacy across diverse soil types and agronomic practices. Moreover, the short shelf life of beneficial microbes poses a significant application challenge. The reported 12-week room-temperature viability of strains like *K. pneumoniae* JAS8 [13] highlights a trait crucial for formulation and distribution logistics.

Addressing the significant biosafety concerns associated with *K*. *pneumoniae* constitutes a critical future research imperative. The comprehensive safety assessment required for any live bacterial application is often resource-intensive, involving substantial financial and temporal investments. Promisingly, studies indicate that key functional agents, such as the extracellular enzymes responsible for degrading zearalenone and crude oil, as well as the heat-stable elicitors from inactivated *K. pneumoniae* SnebYK that induce soybean resistance to atrazine, can operate independently of viable cells. These findings indicate that the key bioactive components may be isolated and applied directly. This strategy could creatively circumvent the human health risks posed by deploying live pathogenic bacteria into open environments. Studies such as the use of a purified azoreductase for dye degradation [81] and the isolation of a phytase to enhance maize root growth [130] demonstrate the feasibility of an enzyme-based approach. Therefore, a crucial future direction involves the identification, characterization, and formulation of these discrete functional components. This shift from whole-cell inoculants to defined “biocatalytic formulations” or “elicitor cocktails” offers a pathway to harness the beneficial traits of *K. pneumoniae* while fundamentally circumventing the risks associated with deploying a live, potentially pathogenic bacterium in agricultural settings. Advancing this strategy, however, is contingent upon a more profound elucidation of the underlying mechanisms of action.

## 7 Conclusions

*K. pneumoniae* can degrade approximately 50 agricultural pollutants, including pesticides, veterinary pharmaceuticals, biological toxins, exogenous pollutants, and agricultural wastes. This degradation offers an economical and environmentally friendly solution for pollution in agricultural production. Additionally, *K. pneumoniae* is an excellent PGPB and biocontrol agent that enhances plant nutrition by fixing nitrogen, solubilizing minerals, and generating plant hormones, thereby promoting plant growth. It also produces antagonistic substances and induces resistance or tolerance for addressing biotic and abiotic stresses. These attributes offer a viable approach to increasing agricultural productivity and sustainability. In parallel, the specific biosafety risks of *K*. *pneumoniae* require careful integration into comprehensive risk assessment and management strategies.

In general, *K*. *pneumoniae* represents a valuable resource for bioremediation, biofertilization, and biocontrol. With further research and biotechnological advances, *K. pneumoniae* holds broad promise for sustainable agricultural development.
